# Evaluating the implementation of online research training and mentorship among early-career family physicians in sub-Saharan Africa

**DOI:** 10.1017/S146342362400063X

**Published:** 2025-01-09

**Authors:** Darcelle Schouw, Robert Mash, Pius Ameh, Bolatito B. Fatusin, Stephen Engmann

**Affiliations:** 1 Division of Family Medicine and Primary Care, Stellenbosch University, Cape Town, South Africa; 2 Department of Family Medicine, Federal Medical Centre, Keffi, Nigeria; 3 Department of Family Medicine, Federal Medical Centre, Abeokuta, Nigeria; 4 Polyclinic/Family Medicine Department, Korle Bu Teaching Hospital-Accra, Ghana

**Keywords:** Academic training, capacity building, distance learning, family physicians, internet use, mentorship, sub-Saharan Africa

## Abstract

**Background:**

Research is needed to improve the performance of primary health care. In Africa, few family physicians conduct research, and therefore an online research training and mentorship programme was developed to build research capacity amongst novice and early career researchers.

**Aim:**

To evaluate the implementation of the AfriWon Research Collaborative (ARC) training and e-mentorship programme in sub-Saharan Africa.

**Methods:**

A 10-module online curriculum was supported by peer and faculty e-mentorship, to mentor participants in writing a research protocol. A convergent mixed methods study combined quantitative and qualitative data to evaluate nine implementation outcomes.

**Findings:**

Fifty-three participants (20 mentees, 19 peer mentors, and 14 faculty mentors), mostly male (70%), participated in the ARC online programme. The programme was seen as an acceptable and appropriate initiative. Mentees were mostly postgraduate students from African countries. Faculty mentors were mostly experienced researchers from outside of Africa. There were issues with team selection, orientation, communication, and role clarification. Only 35% of the mentees completed the programme. Alignment of mentoring in teams and engagement with the online learning materials was an issue. Costs were relatively modest and dependent on donor funds.

**Conclusion:**

Despite many challenges, the majority of participants supported the sustainability of the programme. The evaluation highlights the strengths and weaknesses of the ARC programme and e-mentoring. The ARC working group needed to ensure better organization and leadership of the teams. Going forward the programme should focus more on developing peer mentors and local supervisory capacity as well as the mentees.

## Introduction

The health status of a population is linked to the quality of its primary health care (Phillips *et al.*, 2020). To improve the performance of primary health care, there is a growing consensus on the need for an evidence base that is driven by research (van Weel, 2011). Many of the primary care disciplines in low- and middle-income countries have little or no research activity (such as nurse practitioners and mid-level workers); however, family medicine (FM) has been attempting to develop its research capacity (R. Mash *et al.*, 2014).

The African family physician makes an invaluable contribution to the delivery of primary health care services in terms of the model of care and systems to improve quality (Mash, 2022a). They enhance the capacity of the workforce, improve health information systems, and innovate with digital solutions to support service delivery. Their contribution results in improved access and utilization of care as well as improved core functions of primary care (first-contact access, coordination, comprehensiveness, continuity, and person-centredness). Furthermore, the roles of the family physician are described as a clinician and consultant to the health care teams in primary health care and primary hospitals (Mash, 2022b) as well as clinical governance, clinical training, and capacity building. Family physicians are expected to perform research during their training and to use these skills as part of clinical governance to improve the quality of care and patient safety. Some family physicians may become clinician-scientists or academics and become more established researchers.

While other specialties focus on specific body organs and diseases, FM often focuses on undifferentiated problems, multi-morbidities, and complex relationships between biomedical, social, and economic factors. This holistic approach to health care delivery and research is what positions the family physician as an important resource to a defined population (Rosser and Kasperski, 1999). Therefore, the body of scientific evidence that derives from this kind of practice reflects the complexity and unique approach of FM, to patient care, service delivery, and population health.

Even though research is important in helping physicians remain responsive to the needs of the population they serve, there is currently a dearth of physicians who have chosen this as a long-term career plan, particularly among young graduates (Kwan *et al.*, 2017). For most FM training programmes in sub-Saharan Africa (SSA), the trainee is required to demonstrate research skills by completing a thesis (Flinkenflögel *et al.*, 2014). However, lack of time, poor supervision, and a high clinical workload have been cited as some of the reasons why FM trainees are unable to acquire research skills and successfully complete their thesis during the training programme (Yakubu *et al.*, 2018). In addition, the absence of established researchers and mentors is another important reason why young family physicians in SSA are unable to acquire research skills or maintain a scholarly culture after their first mandatory research project (Yakubu *et al.*, 2018).

Building research capacity is defined as ‘equipping individuals, institutions, organizations and nations, to be able to define and prioritize health problems, develop and scientifically evaluate solutions, share and apply the knowledge gained’ (Lansang and Dennis, 2004). More recently, researchers, after conducting a conceptual review of the literature, expanded the definition of research capacity building (RCB), to include a financially sponsored, multifaceted, and robust effort aimed at expanding research know-how or attaining research goals that are enduring, with a vision of social development (Cooke, Gardois and Booth, 2018). This definition more closely reflects the challenges of conducting RCB activities in low- and middle-income countries, which often includes a lack of available research mentors.

There are various definitions of mentorship, which reflect the various disciplines involved. These include professional development in the context of business and corporate culture, child and adolescent development in the context of teaching, as well as clinical development in the context of medicine (Gagliardi *et al.*, 2014). Mentorship is defined as ‘a dyadic association within which someone obtains guidance, instruction and/or caution, usually from a more advanced learner, as a reciprocal, supportive activity employing educational and social learning principles to strengthen the acquisition of new knowledge, skills and attitudes’ and as ‘a dynamic, reciprocal relationship in a work environment between an advanced career incumbent (mentor) and a beginner (protégé), aimed at promoting the development of both’ (Shrestha *et al.*, 2009; Pillon, 2013; Gagliardi *et al.*, 2014).

The advantages of the mentorship relationship are accruable to both the mentor and mentees and include career advancement and psychosocial benefits for the mentee and job satisfaction for the mentor (Gagliardi *et al.*, 2014). Despite these benefits, problems encountered in mentoring include difficulty in initiating the relationship, especially in the context of physical separation and work timetable clashes (Pillon, 2013; Gagliardi *et al.*, 2014). The attempt to overcome these barriers in mentoring, with advances in technology and communication, including the internet, has led to the development of e-mentoring.

E-mentoring is defined as ‘a naturally occurring relationship or paired relationship within a [programme] that is set up between a more senior/experienced individual (the mentor) and a lesser skilled individual (the mentee), primarily using electronic communications, and is intended to develop the skills, knowledge and confidence of the lesser-skilled individual to help him or her succeed’ (Shrestha *et al.*, 2009). E-mentoring could be seen as simply a way around time and distance constraints, experienced by those wishing to establish mentoring relationships. E-mentoring potentially offers additional benefits over face-to-face mentorship relationships, including reduced social interference, communication flexibility, and greater assertiveness (Shrestha *et al.*, 2009).

Online learning platforms, which may include an e-mentorship component, have the potential to be a powerful tool for RCB, due to their attractiveness for overcoming distance and time constraints. Others have reported drawbacks of using an online learning platform, which included limited discretionary time, lack of technical know-how, and perceived lack of involvement of other participants (Dini *et al.,* 2017).

In 2019, the AfriWon Research Collaborative (ARC) programme was designed and funded to provide online research training and e-mentorship in order to increase research activity among members of a young family physicians’ professional network in SSA, the AfriWon Renaissance or ‘AfriWon’. The ARC pilot programme was initially developed, implemented, and evaluated by an ARC working group. The ARC working group included a family practitioners training group and administrators, within SSA and Boston University, who met bi-weekly. In addition, an ARC advisory group met quarterly to support and guide the working group. The advisory group included members of the World Organization of Family Doctors (WONCA), AfriWon, and the ARC working group.

This pilot programme was found to be both feasible and acceptable and resulted in 4 of the 10 pilot participants completing full research proposals, with all participants having completed more than half of the curriculum deliverables (Mcguire *et al.*, 2021). However, further implementation research was needed to explore the costs and feasibility of the programme with larger numbers of participants, from more diverse contexts in the region and with limited communication infrastructure (Glasgow, Vogt and Boles, 1999; Peters *et al.*, 2013).

A new ARC cycle was implemented in 2021, made possible by funding obtained via the Primary Care and Family Medicine network (Primafamed), based at Stellenbosch University, and the Primary Health Care Research Consortium, of which Primafamed is a member. This evaluation was needed to determine optimal implementation strategies and scale up efforts for the ARC training programme and e-mentorship.

The aim of the study was to evaluate the implementation of the ARC training and e-mentorship programme for novice and early-career FM researchers in SSA. The evaluation focused on a range of implementation outcomes: adoption, appropriateness, acceptability, feasibility, fidelity, coverage, costs, effects, and sustainability of the intervention.

## Methods

### Study design

A convergent mixed methods study combined qualitative and quantitative data to evaluate the key implementation outcomes (Proctor *et al.*, 2011). The evaluation focused on the implementation of the programme in SSA over a 6-month period from May to October 2021. The outcomes were defined as:

1. *Acceptability:* Why did participants and mentors perceive that it was worth doing? What were the factors for and against this?
2. *Adoption:* Why did participants and mentors decide to collaborate and adopt the intervention? What were the key factors they considered in making this decision?
3. *Appropriateness:* Did participants perceive that the intervention was fit for purpose
4. *Feasibility:* When it was implemented what happened? How feasible was it to implement successfully? What were the factors that enabled and hindered implementation?
5. *Fidelity:* How was the programme modified or customized to make it work? Why was this necessary? What were the implications of this?
6. *Coverage:* How many mentees and mentors applied, were enrolled, and completed the intervention? How many mentors participated?
7. *Costs:* What were the set-up and operational costs?
8. *Sustainability:* Should this be sustained? What are the future opportunities and threats to the sustainability of the intervention? Can implementation be taken to scale?
9. *Effects:* What was the effect of the intervention on the mentees’ research capability? What was the effect on the mentors’ ability to mentor?

### The ARC training and mentorship programme

The programme included three types of participants: mentees, peer mentors, and faculty mentors. Mentees were novice researchers, either registrars in training or newly qualified family physicians without any publications in peer-reviewed journals. Peer mentors were family physicians with at least one publication in a peer-reviewed journal. Faculty mentors were more experienced faculty-level family physicians with at least five publications. ARC invited mentees and mentors to register for the programme and each person completed an online registration form. Participation was free, and there was no remuneration for the mentors.

Mentees were asked about their research interests, their availability, and which country they were from. The ARC working group used a scoring system, with more marks assigned to mentees applying from countries with limited supervisory capacity and new FM training programmes. Peer mentors and faculty mentors were selected according to their interest in the programme, expertise, and research field. If possible, they were matched with mentees who had similar interests.

**Figure 1.**
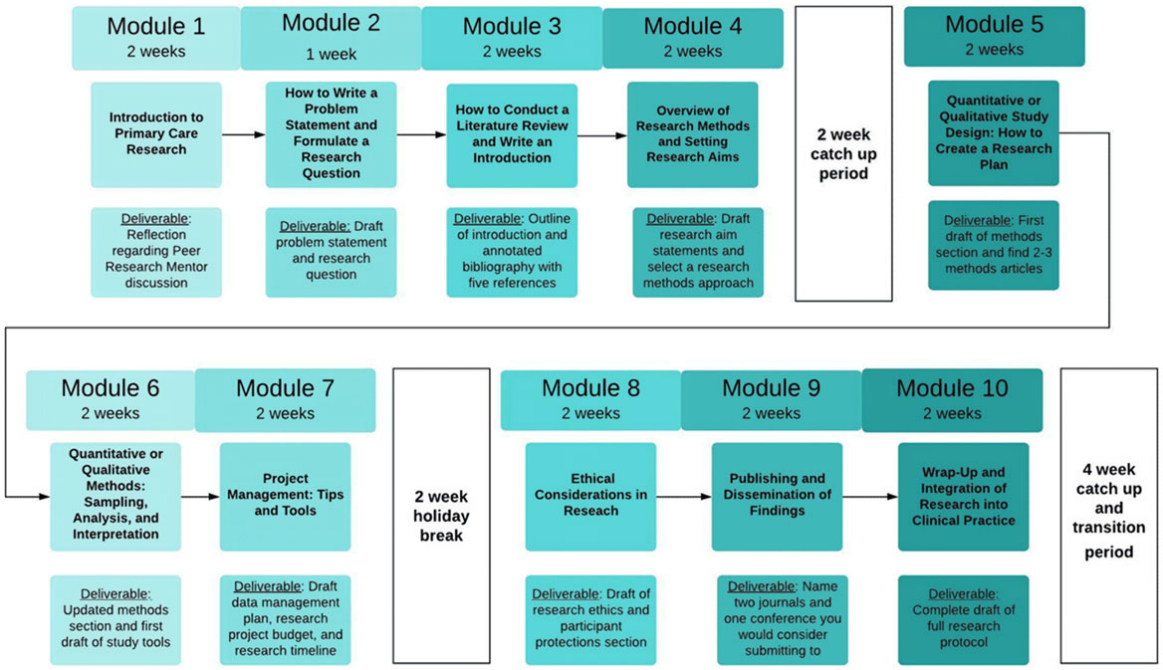
The 10-module ARC curriculum.

The ARC programme provided an online curriculum and virtual e-mentorship, over a 6-month period. The programme ran from May to October 2021 with 20 mentee participants. The curriculum had 10 modules and was designed to guide participants step by step through writing a research proposal (Fig. 1). An online graduation ceremony was held during which mentees received a certificate following completion of the 10 modules.

Each module contained one or more recorded lectures, supplemental reading materials, short assignments or ‘deliverables’, and was accessed via Google Classroom. All mentees were provided with an e-book on ‘How to do Primary Care Research’ (Goodyear-Smith and Mash, 2018).

Peer mentors provided mentorship to mentees, whilst faculty mentors provided mentorship to peer mentors and mentees. The ARC working group divided the participants into 10 mentoring groups. Each group had a faculty mentor, two peer mentors, and two mentees. The ARC programme provided guidelines for communication modes, frequency, and mentorship. The recommended mentoring style was derived from motivational interviewing (Tollefson *et al.*, 2013). WhatsApp groups were created, and participants were encouraged to check in regularly. Members of the ARC working group were assigned to each of the 10 mentoring groups to act as liaisons between the mentoring groups and the ARC working group and to help resolve challenges that may arise.

### Collection and analysis of qualitative data

Exploratory, descriptive semi-structured interviews with participants explored the acceptability, appropriateness, adoption, feasibility, fidelity, sustainability, and effects of the programme.

Twenty mentees, 19 peer mentors, and 14 faculty mentors participated in the programme over 6 months. The intention was to conduct individual interviews with 10 mentees, 5 peer mentors, and 5 faculty mentors as well as conduct a focus group interview with the ARC working group. If data saturation was not achieved in the last two interviews with mentees and mentors, then additional interviews could be conducted. As all mentees and mentors had equal value in terms of sharing their experiences and perspective they were selected randomly. The entire ARC working group was selected for a focus group interview. Participants were invited via email once the programme had ended, and if they did not respond, the next randomly selected person was invited.

An interview guide ensured that all the implementation outcomes were explored: adoption, appropriateness, acceptability, feasibility, fidelity, and sustainability. In the opening question, respondents were asked about their experience of the online training and mentorship. Respondents were also asked about the effect of the programme on their research expertise or ability to mentor. The interview guide listed a variety of open questions, which were used to explore different implementation outcomes. DS recorded semi-structured interviews in English, via Zoom for over 30–60 min.

Audiotapes were transcribed verbatim and checked for errors. Transcripts were analysed deductively using Atlas-ti software to identify themes that related to the implementation outcomes. The researcher used the framework method below to analyse the data (Ritchie, 1994):

1. Familiarization: Reading the transcripts and identifying key issues that should be coded.
2. Coding index: Creating an index of codes and organizing them in categories.
3. Coding: Coding all data according to the coding index.
4. Charting: Gathering all data on the same code or category together in a chart.
5. Interpretation: Interpreting each chart for the key themes, range of ideas and experiences within a theme, and any relationships between themes.

The different data sources and perspectives were triangulated in the analysis, which contributed to its credibility. RM participated in the construction of the coding index and interpretation.

DS was employed as a researcher in the Division of Family Medicine and Primary Care at Stellenbosch University with experience of implementation science and qualitative studies. She is a biokineticist by background and was not involved in the ARC programme prior to the evaluation. RM is an established researcher in the same Division and was involved in funding and supporting the ARC programme as a member of the advisory group. He also participated as a faculty mentor in the programme.

### Collection and analysis of quantitative data

#### Evaluation of coverage

Data were obtained from the ARC registration forms on the mentees and mentors as well as from Google Classroom on the mentees’ participation. The data provided information on demographic characteristics of mentors and mentees (age and sex); geographic spread; job roles and qualifications; reasons for participating; number of people accessing Google Classroom per month; number of mentees accessing each module and the number of mentees submitting assignments. Data were analysed descriptively for the mentors and mentees using the Statistical Package of Social Sciences (SPSS) version 27.

#### Evaluation of effect of research training and e-mentorship

An online descriptive survey was administered to the participants at the end of the 6 months to evaluate the effects of the programme. One questionnaire was designed for the mentees and another for the mentors. For mentees, the questions asked about their research aim; study design; stage of completion of the research proposal and ethics approval; change in knowledge with regard to the 10-module topics; change in confidence/motivation with regard to primary care research; quality of mentorship in terms of guidance, communication, feedback, and expertise; frequency of interaction with their mentors; satisfaction with the ARC programme and feedback on what could be improved.

For mentors, the questions asked about their development as a mentor in terms of guidance, communication, feedback, and expertise; change in confidence/motivation with regard to primary care research; change in confidence/motivation with regard to mentoring others; satisfaction with the ARC programme; and feedback on the programme and what could be improved. The questionnaires were designed by the researchers, validated by the ARC leadership, and administered online via REDCap.

All quantitative data were imported into the Statistical Package for Social Sciences (version 27) and analysed descriptively. Frequencies and percentages were used for categorical data, while means and standard deviations or medians and interquartile ranges were used for numerical data, depending on its distribution.

#### Evaluation of set-up and operational costs

Evaluation of costs focused on the incremental set-up and operational costs for ARC to run the programme over 6 months.

### Integration of analysis

The qualitative and quantitative findings were integrated to give a more complete evaluation of the implementation outcomes and objectives. Where possible joint displays were created or the qualitative interpretation presented alongside the quantitative findings in a table.

## Findings

Thirteen qualitative interviews were conducted; 11 individual interviews with 4 mentees, 1 peer mentor and 6 faculty mentors; and 2 small group interviews covering 9 members of the ARC working group. Only 17 out of the 53 people invited completed the questionnaire (32%) and included 4 mentees, 7 peer mentors, and 10 faculty mentors. This section integrates the findings derived from both quantitative and qualitative data and presents them according to the implementation outcomes.

### Adoption, Acceptability. Appropriateness (adoption)

On registration, mentees and mentors gave a variety of reasons for adopting the programme on their forms as shown in Table 1. These reasons are illustrated by quotations from the interviews in a joint display.

**Table 1.** Participants’ reasons for participating

Reasons for participating	n	Supporting quotations
**Faculty mentors (N = 14)**		
To increase research capacity and provide mentorship	6	*‘I wanted to support, the idea of having sort of an African support system for new researchers or, you know, researchers to get going. I mean, it’s something I hold close to my heart’. (Faculty Mentor)*
Share experience and transfer skills	3	
Participated in first ARC cycle	2	
Funder	1	
Meet and collaborate with early-career researchers in SSA	3	
Understand primary care in other countries	1	
		*‘It’s such a really great concept, you know, sort of online mentoring for novice and early career researchers in sort of Family Medicine/Primary Care in the region. There’s a big need for it, I think. So the idea is great’. (Faculty Mentor)*
**Peer mentors (N = 19)**		
Enhance skills and learn more about clinical research	13	*‘I wish to mentor young family physicians in the field of research while improving my research skills and contributing to family medicine research in Africa’. (Peer Mentor)*
Provide mentorship, share expertise	7	
Collaborate and build connections	5	
Personal research development	2	
Interested in public health	1	
Participated in first ARC cycle	1	
		*‘I love research and after my residency training program, I have come to appreciate the richness research brings to practice. Therefore I would love to encourage and mentor other family physicians who are interested in research to put in their best’.(Peer Mentor)*
**Mentee (N = 20)**		*‘I believe it will offer me the needed push to really engage in clinical research as part of my professional career. I believe it is a great networking platform for career enhancement. I will have the opportunity to learn and be guided by astute medical professionals’. (Mentee)*
		*‘I would like grow as a researcher in the field of family medicine, the speciality is still in its infancy in Botswana and I believe research will work towards building it. I also would like to branch towards academic side of family medicine’. (Mentee)*

The online and virtual nature of the programme meant that faculty mentors could be global even if the peer mentors and mentees were from SSA. The COVID-19 pandemic also encouraged people to engage virtually:

*‘It’s a good programme, especially considering the current COVID-19 pandemic, it was an opportunity to learn and also to connect the network with experts, and also peers from other countries across the continent’. (ARC working group)*

Mentors and the ARC leadership clearly saw the need for the programme and its potential to build a community of practice. Those that reviewed the online curriculum found it to contain useful and relevant material:

*‘When I joined ARC as part of my practice, I joined when I saw that we were trying to help build research capacity in physicians in Sub Saharan Africa I said, wow, this is exactly what I want to do. And so that is what sort of influenced me to continue and still keeps me in this group to try and make research in Sub Saharan Africa better and I think is an awesome thing that we are doing’. (ARC WG)*

### Coverage

Fifty-three participants enrolled for the programme of which 20 were mentees, 19 were peer mentors, and 14 faculty mentors (Table 2). Two of the mentees were family physicians and 18 mentees were resident doctors. The majority of the participants (70%) were male. Faculty mentors came from nine countries, mostly outside of SSA. Peer mentors came from six countries, mostly from SSA. Mentees came from six countries in SSA.

**Table 2.** Participants in the programme

Participants	Male n (%)	Female n (%)	Countries reached
Faculty mentors (N = 14)	13 (93)	1 (7)	Netherlands, Denmark, South Africa, United Kingdom, New Zealand, Ghana, India, Turkey, Australia
Peer mentors (N = 19)	12 (63)	7 (37)	Nigeria, Brazil, Democratic Republic of Congo, RC, Croatia, Ethiopia, Lesotho
Mentees (N = 20)	12 (60)	8 (40)	Ghana, Nigeria, Democratic Republic of Congo, Malawi, Botswana, Zimbabwe
Total (N = 53)	37 (70)	16 (30)	

### Feasibility and fidelity to the programme design

There was a 3-week period of preparation between the recruitment of participants and the start of the programme. Mentors and mentees were organized into 10 teams. An orientation programme was offered by the ARC working group to explain how the programme was meant to work, but only 10 out of the 53 participants attended the session. Therefore, the slides from the orientation session were sent to the participants. Although peer mentors were meant to lead and initiate the group interactions, several were not clear about their intended role, a few dropped out, and some inadvertently excluded the faculty mentors. Some faculty mentors did not receive any further feedback or communication on their roles in the teams. Lack of communication between peer and faculty mentors was a challenge to successful implementation. This meant that many teams were dysfunctional and without effective leadership:

*‘And I see the orientation, it is one of the very key aspects, or important part of the whole mentorship programme, because what I observed was that, especially the peer mentors, because some of them didn’t turn up at the orientation. They were not too sure what their role was, in the whole ARC programme’. (ARC WG)*

*‘The barriers I would say the barrier, one major barrier we have is the peer research mentors not communicating with the faculty research mentors, because as senior colleagues they are expecting the peer mentors to get in touch, to engage them. But they were not engaged as expected’. (ARC WG)*

WhatsApp groups were supposed to be created to facilitate the mentorship component of the programme and to respond to problems experienced by the participants, to allow the participants to check in at regular intervals, and in most cases, the groups were created by the ARC working group. The WhatsApp groups proved to be somewhat successful in overcoming communication challenges and providing feedback for a small number of participants.

The ARC working group arranged to have meetings with the mentees every 2 weeks to monitor activities; however, participants were not always available and did not always respond to messages posted in the WhatsApp groups. Personal or family challenges, high workloads, exams, competing interests, and time constraints were cited as reasons for mentees not completing the programme or responding to the WhatsApp messages. For some of the mentees, the programme was deemed too demanding. Members from the ARC working group and faculty mentors indicated that some of the mentees were not sufficiently motivated, which resulted in them not attempting some of the modules. Mentees needed encouragement and support from the mentors, and some mentors felt that mentees should have been better selected:

*‘I felt the barrier on my part, as somebody who has got a full-time job was that sometimes I’ll put, I will set targets for myself to say, okay, on such and such a day, I should do ABCD or I should attend to assignment ABCD. But then, sometimes you are required even to go like to the field. Yes, where maybe access to the internet is not there. Or sometimes even if access is there, you just don’t have that time to sit on the computer and concentrate on your work. So that to me, I found a barrier. So much so that I found myself being behind my own schedule’. (Mentee)*

Some of the mentees found the e-mentoring programme to be organized and well structured and were able to follow and complete the modules with the help of their mentors. The programme was flexible and allowed mentees to catch up on modules they had not completed in the time frame expected. Participants who could not follow the prescribed timeline were allowed to come up with their own deadlines:

*‘I think the online training is actually quite good, because there’s regular communication with the organisers as well as the administrators. And there’s that personal touch where you have your mentor, who’s, you know, you’re talking to almost on a daily basis. My peer mentor, we were talking on WhatsApp almost every day’. (Mentee)*

*‘The mentees, were able to engage with the modules. I can recall from my last meeting, that we have, like a log for the mentees to see how they are progressing through the modules. So I think we have like three or four who have completed modules and we have some of them who are like halfway through the modules, while, I think there was a mentee that was inactive from the beginning nothing, so we tried getting in touch but we could not get him to start’. (ARC working group)*

Engaged peer mentors were able to gain experience through the online mentorship process, especially through the modelling of interaction and feedback and indirect coaching provided by the faculty mentors:

*‘So the peer mentor is getting experience of research supervision. And the mentee is getting experience of course, in this case, writing a proposal and so it’s sort of like a triad. And I think that did happen well, you could ask my peer mentor, but, I mean, I did find that some of the feedback from the peer mentor, in my view, was not correct’. (Faculty Mentor)*

Some of the more functional teams met virtually using video conferencing such as Zoom, and this appeared to build their relationships and clarify goals and expectations. In addition, these teams might also make use of online tools such as Google Docs to share emerging proposals and collaborate on providing comments and feedback.

However, according to most of the faculty mentors, the mentoring programme was disorganized and chaotic, with few of them receiving communication on their roles, responsibilities, or the expected outcomes. Faculty mentors were unable to access Google Classroom as the free version restricted the number of participants. Therefore, they did not have access to the training material online. Some faculty mentors also found the scheduling of check-in meetings on a Sunday to be inappropriate, making it difficult for them to attend, due to family commitments. Some faculty mentors experienced frustration at the lack of communication from the ARC working group and mentees, which resulted in them withdrawing from the programme:

*‘So, you know, they should really have a system where all of the mentors and all of the mentees can see the educational programme. Likewise, so, as a mentor, I wasn’t really aware of the content of the programme. But also, I wasn’t really aware of the process’. (Faculty mentor)*

*‘I don’t think it was that successful. As I’ve said, I think, I think, you know, the people who are coordinating things from the centre needed to be a lot more proactive in communicating with people, checking up how things are going, sorting out any misunderstandings, or, you know, making sure that people do connect’. (Faculty Mentor)*

Mentees could submit their assignments online via a link that was provided to them. However, there were limitations in submitting the work via the link:

*‘Because you’re actually given a link to say after you’ve done your work, and you submit it using this link. And when you do that, you have the impression to say they have received my work and then you don’t get the feedback, so to me that was a challenge. But also the other challenge is other than submitting our work but, you know, you would want sometimes maybe to ask a question, through a platform, and then you realise that you ask and then the response is not forthcoming’. (Mentee)*

Faculty and peer mentors completed a self-evaluation of their mentoring activities as shown in Table 3. The majority of mentors that responded to the questionnaire believed they were available, friendly, and supportive. They felt that they were committed to and interested in the research process, were affirming of the mentee, and created an environment where the mentee was comfortable to raise issues. Mentors reported that they assisted with selecting and refining the topic, planning the research, shared new ideas, and provided constructive comment and feedback. They thought that they helped the mentee to be self-directive in their work and learning. Faculty mentors, compared to peer mentors, were significantly more likely to report creating a learning environment, providing constructive comment and feedback, and support self-directed learning.

**Table 3.** Self-evaluation of faculty and peer mentors

	All mentors N = 17 n (%)	Peer mentors N = 7 n (%)	Faculty mentors N = 10 n (%)	*p*-Value
**Mentors assisted with:**	**Agree**	**Agree**	**Agree**	
I was available for discussions/consultations when needed	12 (71)	6	6	0.11
Being friendly, supportive, and helpful	12 (71)	6	6	0.52
Refining topic selection and clarification	11 (65)	6	5	0.28
Providing affirmation and motivation	11 (65)	6	5	0.31
Showing interest in research	11 (65)	6	5	0.31
Showing commitment to the research	10 (59)	6	4	0.16
Creating an atmosphere in which mentee felt comfortable raising issues	10 (59)	6	4	0.12
Planning of research	9 (53)	6	3	0.08
Providing encouragement to work in self-directed manner	9 (53)	6	3	0.04
I engaged in constructive discussions with regard to the proposal	9 (53)	6	3	0.03
I provided constructive feedback for work delivered	9 (53)	6	3	0.03
I provided new ideas for the research	9 (53)	6	3	0.43
Creating a healthy challenging environment	8 (47)	5	3	0.02
Finding literature in the area of research	7 (41)	5	2	0.96

The feedback of faculty and peer mentors on the positive and negative aspects of mentoring are summarized in Table 4. Their qualitative comments have been categorized and quantified. The commonest positive experience was the opportunity to provide constructive feedback and guidance to mentees, while the commonest negative experience was the lack of interaction and contact with the mentees.

**Table 4.** Positive and negative aspects of monitoring by mentors

	All mentors N = 17 n (%)
**Positive aspects**	
Providing helpful feedback and guidance	6 (35)
No comment	5 (29)
Having African support and meeting people	2 (12)
Orientation meeting	1 (6)
**Negative aspects**	
Little to no contact from mentee	6 (35)
No comment	5 (29)
No mentee allocation	4 (23)
Disorganized and delays in initiating contact	4 (23)
Difficulty working with mentees who are not motivated	1 (6)

### Costs

Table 5 lists the costs involved and amounted to R128,539 (US$7841). Each mentee received an e-book and access to the Google Classroom. ARC used the free version of the Google Classroom, which restricted the numbers enrolled. Two Zoom licences enabled group interaction by the working group and meetings with the mentors and mentees. Four members of the ARC working group received honorariums, including the programme director, mentorship director, and two deputy directors. In total, this was the equivalent of 20% of a family physician for 6 months, shared between the four people. The mentors’ time was volunteered, and mentors and mentees paid for their own data costs.

**Table 5.** Set-up and operational costs

Type of costs	ZAR	**$US** ^*^
**Set-up costs**		
e-books	10 946	667
Zoom account licences	2325	142
**Operational costs**		
Administrative support by Primafamed	28 000	1708
Mobile data for ARC working group	2268	138
ARC working group honorariums	85 000	5185
**TOTAL**	**128 539**	**7841**

* 1 ZAR = 0,061 US$ as at 14 August 2022.

### Sustainability

All respondents agreed that despite the challenges, the programme was worth doing and should continue:

*‘I still think the concept is great. It needs to be improved’. (Faculty Mentor)*

*‘I think it’s sustainable provided you do it properly. I think it’s a low-cost initiative, it has a lot of value, cost effectiveness, especially for African researchers, who are without support’. (Faculty Mentor)*

In order to be more sustainable, the organizers need to ensure that the programme is better coordinated, with clear concise communication of roles and outcomes. Respondents felt that selection of mentees should be improved to ensure they were motivated and had the correct expectations. More funding would be needed for future cycles to cover the existing costs but also to expand access to the Google Classroom and the reach of the programme. Currently, mentoring ends with the writing of the proposal, but a more ongoing relationship with the mentee might extend to the research itself and even to a shared publication, which would be an incentive for the mentors:

*‘So I think I would recommend to, to refine the programme, to restructure left and right, to have really good entrance interviews with the participants of what they want and how serious they want to engage in this. And yes, I would love the programme to continue to survive’. (Faculty mentor)*

*‘But the mentoring could continue, potentially. And in fact, you know, if the mentors went on a journey with those researchers and got a publication out of it, then that would be a tangible benefit for the mentors’. (Faculty mentor)*

### Effects

**Figure 2.**
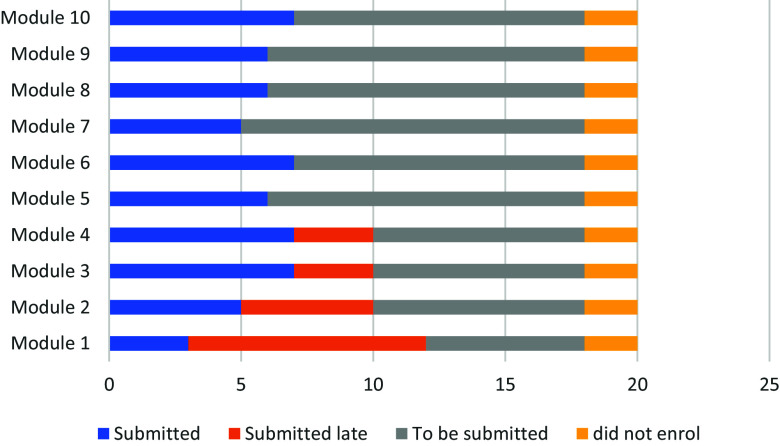
Number of online modules completed by mentees (N = 20).

Of those that enrolled a number were completely inactive in the programme. This included 8/14 (57%) faculty mentors, 4/19 peer mentors (21%), and 6/20 (30%) mentees. The remainder were active to some extent during the programme. At least half of the mentees submitted the work for modules 1–4, but after this participation dropped (Fig. 2).

Only 7/20 (35%) of the mentees, successfully completed the online training programme and submitted a final draft proposal. Only one mentee completed all 10 modules in the 6-month time frame. Another seven mentees (35%) participated in some of the modules but did not complete a research proposal. Mentees that completed the programme learnt how to write a proposal and reported being more confident in conducting research:

*‘I can say I’m more confident now drafting a research proposal, doing a literature review, I think, I would say I am more confident and I’m looking forward to you know writing more proposals. I noticed that also following through with my research, so it was a good experience for me’. (Mentee)*

Engaged peer mentors were also able to gain experience through the online mentorship process, especially through the modelling of interaction and feedback and indirect coaching provided by the faculty mentors:

*‘So the peer mentor is getting experience of research supervision. And the mentee is getting experience of course, of, in this case, writing a proposal and so it’s sort of like a triad. And I think that did happen well, you could ask my peer mentor, but, I mean, I did find that some of the feedback from the peer mentor, in my view, was not correct’. (Faculty mentor)*

## Discussion

### Summary of key findings

Overall, the programme had good acceptability, was seen as appropriate, and was adopted by a cohort of mentees and mentors. The reach was limited and was mostly to postgraduate students. Feasibility and fidelity were also problematic as only a small proportion of mentees completed the programme and many mentors also dropped out. Despite this, the costs were minimal per mentee (US$392), and the respondents felt the programme was worth continuing.

**Figure 3.**
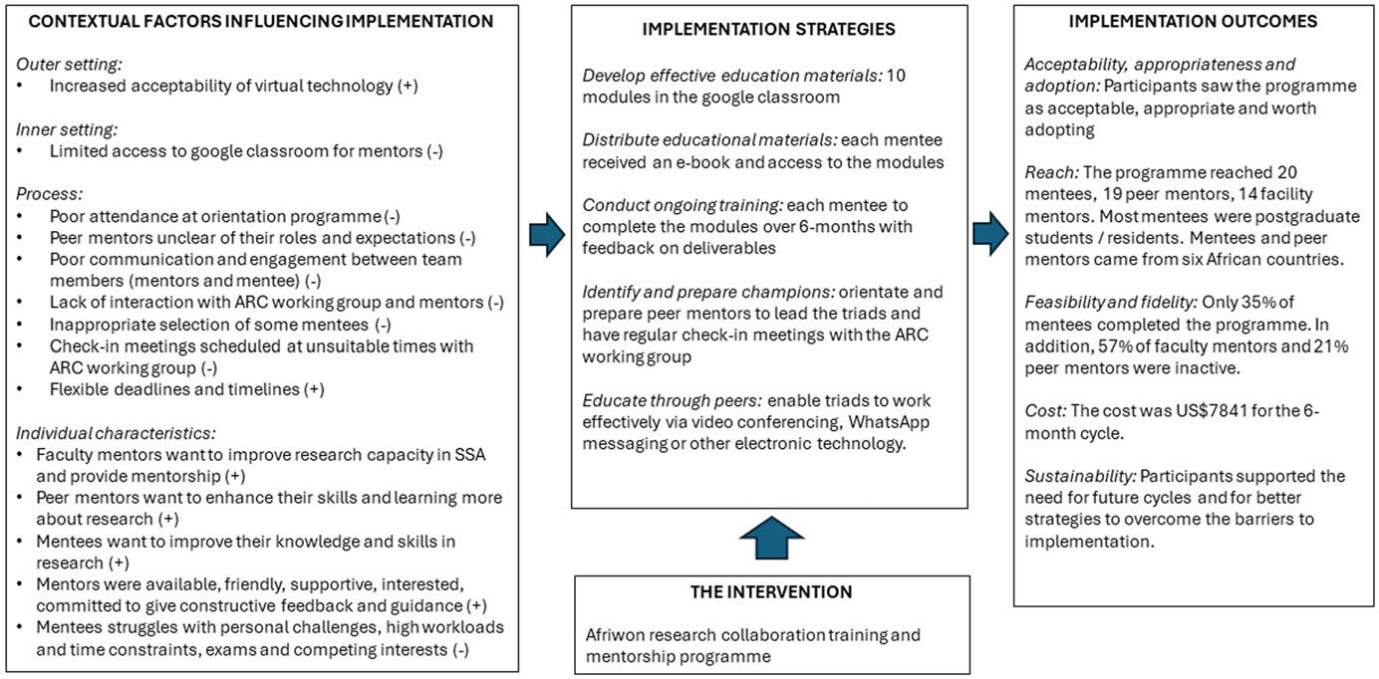
Summary of key findings in an implementation research logic model.

The key findings are summarized in Fig. 3 using the implementation research logic model (Smith, Li and Rafferty, 2020). The contextual factors that acted as barriers or enablers to implementation are summarized using the constructs from the consolidated framework for implementation research (Rapport, Clay-Williams and Braithwaite, 2022). Many of the enablers related to the personal characteristics of the mentors and mentees, while most of the barriers related to process issues. The implementation strategies used were identified in the findings and classified according to a recognized typology (Powell *et al.*, 2012). Strategies were almost all in the educational and planning domains. The strategies need to be revisited to overcome the process barriers in future cycles.

## Discussion of key findings and implications

Most of the mentees in the ARC programme were postgraduate students in FM training programmes who were required to complete a research study for their Master’s degree or Fellowship (Mcguire *et al.*, 2021). They were therefore enrolled with a higher education institution, but in many countries these institutions lacked effective postgraduate research supervisors and students looked to ARC for assistance. Postgraduate supervisors may lack research expertise and experience and also be hampered by competing demands on their time from teaching and clinical practice (Jenkins *et al.*, 2020; Ameh *et al.*, 2022). In effect, therefore, the mentors were standing in for or supplementing the official supervisors of these students.

If the need is for ARC to supplement or support training programmes that lack sufficient supervisory capacity, then this could be done in a more explicit and intentional way. For example, the ARC programme could enter into agreements with specific institutions to assist their students and to help build supervisory capacity. The ARC programme could then be better integrated with the student’s training programme and timetable and focus, not only on writing the research proposal, but maybe more importantly on strengthening the local supervisor. This implies that in these situations the peer mentor should be the local supervisor from the student’s institution. Institutions though need to also ensure that the research process is supported by the availability of small-scale funding, efficient and constructive ethics committees, access to library resources and databases as well as software to enable data analysis (Ameh *et al.*, 2022). Mentoring and supervision alone are not sufficient to ensure success.

The number of qualified family physicians joining the programme as mentees was small, and most African family physicians in clinical practice struggle to engage with formal research projects (Mash *et al.*, 2014). A recent review of the contribution of family physicians to African health systems noted that many family physicians demonstrated the use of the research toolkit in clinical governance and more informal workplace-based evaluations, with a focus on quality improvement (Mash, 2022). The development of practice-based research networks and collaboration with more academically minded family physicians may also be a feasible model to enable research in clinical practice (Mash, 2020). It may also be helpful for departments to agree on a research agenda and to help family physicians integrate research into their service delivery.

The creation of effective mentor–mentee teams requires attention to the building of relationships and a common understanding of the ARC programme. Mentor–mentee relationships should be collaborative and avoid the hierarchical nature of supervision and power dynamics that often characterizes African higher education (Jenkins *et al.*, 2020). Teams that made use of the available technology to build relationships and understand each other’s expectations and context appeared to perform better (Mcguire *et al.*, 2021). The ARC working group should ensure that teams meet and engage early on in the programme. The previous evaluation of the ARC program also noted the linkage between effective communication in the teams and success with the programme, and that confusion over roles was a problem (Buadu *et al.*, 2021; Mcguire *et al.*, 2021). In particular, the leadership of the teams needs to be clarified and peer mentors seemed to struggle with this. Consideration should be given to a stronger role for the faculty mentors, who appeared very committed to the success of the programme. The programme should also give more guidance on the mentoring relationship between the faculty and peer mentor as developing the peer mentor is equally important. Previous studies in the same context have also suggested that the programme could incentivize mentors by helping them prepare for doctoral studies and including them in any publications that eventually arise from the mentoring (Jenkins *et al.*, 2020; Mcguire *et al.*, 2021).

The separation of the online curriculum and modules from the mentoring process was also a problem as mentors were not aware of what was taught or happening in the Google Classroom. Many of the peer mentors might also have benefited from access to the resource materials. Previously, mentees have found the online resources to be very useful (Mcguire *et al.*, 2021). Additional access to the Google Classroom would need a licence and additional costs.

The ARC programme only focuses on the development of a research proposal, but of course, the research process requires attention to data collection, analysis, and report writing as well as publication and knowledge translation (Goodyear-Smith and Mash, 2018). To fully equip peer mentors and mentees, the programme may need to expand the mentoring relationship to include these other aspects of the research journey. Currently, the ARC organizes ‘work in progress’ meetings every month, after the programme itself has ended, to allow participants to present their ongoing research work and receive additional feedback from faculty mentors.

The ARC programme is currently free to the participants and relies on grant or donor funding. At present, it seems that faculty mentors are willing to volunteer their time in a commitment to developing FM in Africa, but this motivation may be eroded if the experience of mentoring is not well organized. The development of a cohort of more competent local supervisors (peer mentors) who can sustain research supervision and who can progress to becoming more experienced researchers should be a more intentional output of the programme. The need for more training in mentorship and postgraduate supervision has previously been noted (Jenkins *et al.*, 2020).

It appeared that the programme adopted the term mentoring to imply a more informal, collaborative, and guiding style and to avoid the concept of supervision, which implied a more formal, authoritarian, and directive style that was also linked to assessment.

### Strengths and limitations

The mixed methods approach to evaluation of the programme allowed triangulation of the quantitative and quantitative findings. Only 4/20 (20%) of the mentees completed the questionnaire and as the response rate was too low to produce valid results, these results were not presented. It appeared that the mentees who did not complete the programme were not willing to participate in the evaluation. The four mentees that were interviewed, therefore, may have had a more positive view. Likewise, only one of the peer mentors was willing to be interviewed and only 7/19 (37%) completed the questionnaire. The viewpoint of the peer mentors, therefore, is under-represented in the findings. Most of the faculty mentors completed the questionnaire and six were interviewed, and therefore this viewpoint is the strongest contributor to the findings. The findings, however, are sufficiently robust as a whole to provide insight into the strengths and weaknesses of the programme. Nevertheless the limitations in terms of generalisability of the quantitative data should be noted. The analysis was also conducted independently of the ARC working group, and this will have added to the objectivity and reduced any social desirability bias.

## Conclusion

The second cycle of the ARC online training and mentorship programme was seen as an acceptable and appropriate initiative and was adopted by a group of mentees, peer mentors, and faculty mentors. Mentees were mostly postgraduate students from African countries, where institutions had limited supervisory capacity. Faculty mentors were mostly experienced researchers from outside of Africa. Only 35% of the mentees successfully completed the programme, and there were issues with team selection, orientation, communication, and role clarification. The ARC working group need to ensure better organization and leadership of the teams. Alignment of mentoring in teams and engagement with the online learning materials was also an issue, particularly for faculty mentors. Costs were relatively modest and dependent on donor funds. Going forward the programme should focus more on developing peer mentors and local supervisory capacity as well as the mentees.

## References

[ref1] Ameh PO , McGuire CM , Van Waes A , Fatusin BB , MacIntyre LS , Lelei-Mailu F, Kodicherla H , Egyirwa Buadu MA , Dankyau M and Yakubu K (2022) Research activity, facilitators and barriers amongst trainee and early-career family physicians in sub-Saharan Africa: a cross-sectional survey. African Journal of Primary Health Care and Family Medicine 14, 1–10. 10.4102/phcfm.v14i1.3367 PMC9257712 35792629

[ref2] Buadu M , van Waes A , Yakubu K , Ameh P , Fatusin BB , Kodicherla H , Jack BW , Scott NA and McGuire CM (2021) Research e-Mentorship for early-career family physicians in sub-Saharan Africa: evaluation of a pilot programme. The Lancet Global Health 9, S27. 10.1016/S2214-109X(21)00135-2.

[ref3] Cooke J , Gardois P and Booth A (2018) Uncovering the mechanisms of research capacity development in health and social care: a realist synthesis. Health Research Policy and Systems 16, 1–22. 10.1186/s12961-018-0363-4. 30241484 PMC6150992

[ref4] Dini L , Galanski C , Döpfmer S, Gehrke-Beck S , Bayer G, Boeckle M , Micheel I, Novak J and Heintze C (2017) Online platform as a tool to support postgraduate training in general practice – a case report. GMS Journal for Medical Education 34, 1–16. 10.3205/zma001136 PMC5704610 29226227

[ref5] Flinkenflögel M , Essuman A , Chege P , Ayankogbe O and De Maeseneer J (2014) Family medicine training in sub-Saharan Africa: south-South cooperation in the Primafamed project as strategy for development. Family Practice 31, 427–436. 10.1093/fampra/cmu014. 24857843 PMC4106404

[ref6] Gagliardi AR , Webster F , Perrier L , Bell M and Straus S (2014) Exploring mentorship as a strategy to build capacity for knowledge translation research and practice: a scoping systematic review. Implementation Science 9, 1–10. 10.1186/s13012-014-0122-z. 25252966 PMC4182766

[ref7] Glasgow R , Vogt T and Boles S (1999) Evaluating the public health impact of health promotion interventions: the RE-AIM framework. American Journal of Public Health 89, 1322–1327. 10474547 10.2105/ajph.89.9.1322 PMC1508772

[ref8] Goodyear-Smith F and Mash B (2018) How To Do Primary Care Research, 1st Edn. London: CRC Press.

[ref9] Jenkins L , McGuire CM , Yakubu K , Ayisi-Boateng NK , Motlhatlhedi K , Ameh P , Fatusin BB , Makwero M and Jenkins LS (2020) Exploring gaps, strategies and solutions for primary care research mentorship in the African context: a workshop report. African Journal of Primary Health & Family Medicine 87, 1–4. 10.4102/phcfm.v12i1.2320 PMC7284154 32501030

[ref10] Kwan JM , Daye D , Schmidt ML , Conlon CM , Kim H , Gaonkar B , Payne AS , Riddle M , Madera S , Adami AJ and Winter KQ (2017) Exploring intentions of physician-scientist trainees: factors influencing MD and MD/PhD interest in research careers. BMC Medical Education 17, 1–16. 10.1186/s12909-017-0954-8. 28697782 PMC5505137

[ref11] Lansang MA and Dennis R (2004) Building capacity in health research in the developing world. *Bull World Health Organ*, 004093, 764–770. PMC2623028 15643798

[ref12] Mash R (2020) Establishing family physician research networks in South Africa. South African Family Practice 62, a5216. 10.4102/safp.v62i1.5216 (Accessed: 22 November 2021). PMC8378057 33179955

[ref15] Mash R (2022a) The contribution of family medicine to African health systems. *African Journal of Primary Health Care and Family Medicine* 14. 10.4102/phcfm.v8i1.1251. PMC5016715 27608670

[ref16] Mash R (2022b) The contribution of family physicians to district health services in South Africa: a national position paper by the South African Academy of Family Physicians. South African Family Practice 64, 1–7. 10.4102/safp.v64i1.5473. PMC8991216 35384681

[ref101] Mash R , Essuman A , Ratansi R , Goodyear-Smith F , Von Pressentin K , Malan Z , Van Lancker M and De Maeseneer J (2014) Africa: African primary care research: current situation, priorities and capacity building. In Primary Health Care around the World: Recommendations for International Policy and Development. CRC Press, pp. 25–32. 10.4102/phcfm.v6i1.758. PMC5326807 26245447

[ref13] Mash R , Essuman A , Ratansi R , Goodyear-Smith F , Von Pressentin K , Malan Z , Van Lancker M and De Maeseneer J (2014) African primary care research: current situation, priorities and capacity building. African Journal of Primary Health Care and Family Medicine 6(1). 10.4102/phcfm.v6i1.758. PMC5326807 26245447

[ref17] Mcguire CM , Fatusin BB , Kodicherla H , Yakubu K , Ameh P , van Waes A , Rhoad E , Jack BW and Scott NA (2021) Implementation of online research training and mentorship for sub-Saharan African family physicians. Annals of Global Health 87, 1–17. 10.5334/aogh.3171. 33598411 PMC7863851

[ref18] Peters DH , Adam T , Alonge O , Agyepong IA and Tran N (2013) Implementation research: what it is and how to do it. BMJ 347, f6753. 10.1136/BMJ.F6753. 24259324

[ref19] Phillips JF , Binka F , Schleiff M , Bawah AA and Awooner-Williams JK (2020) *Achieving Health for All: Primary Health Care in Action Achieving Health for All*. 10.1353/book.77991.

[ref20] Pillon S (2013) Mentoring in a digital age. Canadian Family Physician 59, 442–444. 23585612 PMC3625091

[ref21] Powell BJ , McMillen JC , Proctor EK , Carpenter CR , Griffey RT , Bunger AC , Glass JE and York JL (2012) A compilation of strategies for implementing clinical innovations in health and mental health. Medical Care Research and Review 69, 123–157. 10.1177/1077558711430690. 22203646 PMC3524416

[ref22] Proctor E , Silmere H , Raghavan R , Hovmand P , Aarons G , Bunger A , Griffey R and Hensley M (2011) Outcomes for implementation research: conceptual distinctions, measurement challenges, and research agenda. Administration and Policy in Mental Health, 38, 65–76. 10.1007/S10488-010-0319-7. 20957426 PMC3068522

[ref23] Rapport F , Clay-Williams R and Braithwaite J (2022) Implementation Science: The Key Concepts. Abingdon: Routledge.

[ref24] Ritchie J and Spencer L (1994) Qualitative data analysis for applied policy research. In Bryman A and Burgess R (eds), Qualitative Data Analysis. London, pp. 173–194. 10.4324/9780203413081.

[ref25] Rosser WW and Kasperski J (1999) Organizing primary care for an integrated system. HealthcarePapers 1, 5–21. 10.12927/hcpap.1999.17444. 12606855

[ref26] Shrestha C et al. (2009) From face-to-face to e-Mentoring: does the “e” add any value for mentors? International Journal of Teaching and Learning Higher Education 20, 116–124.

[ref27] Smith J , Li D and Rafferty M (2020) The implementation research logic model: a method for planning, executing, reporting, and synthesizing implementation projects. Implementation Science 15, 1–12. 32988389 10.1186/s13012-020-01041-8 PMC7523057

[ref28] Tollefson DR , Lundahl B , Moleni T , Burke BL , Butters R , Butler C and Rollnick S (2013) Motivational interviewing in medical care settings: a systematic review and meta-analysis of randomized controlled trials. Patient Education and Counseling 93, 157–168. 10.1016/j.pec.2013.07.012. 24001658

[ref29] van Weel C (2011) The impact of research in primary care and family medicine: the Thomson Reuters Web of Science Subject Category ‘Primary Health Care’. Family Practice 28, 239–240. 10.1093/fampra/cmr021. 21602287

[ref30] Yakubu K , Colon-Gonzalez MC , Hoedebecke K , Gkarmiri V , Hegazy NN and Popoola OO (2018) ‘Meeting report: “How do I incorporate research into my family practice?”: reflections on experiences of and solutions for young family doctors. African Journal of Primary Health Care and Family Medicine 10, e1–e6. 10.4102/phcfm.v10i1.1640. PMC5913785 29781695

